# Analysis of the acute-phase protein response in pigs to clinical and subclinical infection with H3N2 swine influenza virus

**DOI:** 10.1111/irv.12186

**Published:** 2013-11-07

**Authors:** Małgorzata Pomorska-Mól, Kwit Krzysztof, Zygmunt Pejsak, Iwona Markowska-Daniel

**Affiliations:** Department of Swine Diseases, National Veterinary Research InstitutePulawy, Poland

**Keywords:** Acute-phase response, H3N2, pigs, swine influenza

## Abstract

**Background:**

Swine influenza (SI) is a contagious, important respiratory disease. Diagnosis of SI is based on the clinical signs, confirmed by the detection of viral RNA or specific antibodies. However, the infection is much more frequent than the disease.

**Objectives:**

The aim of study was to investigate the kinetics of acute-phase protein (APP) response during subclinical and clinical influenza in pigs. The utility of APP measurements in identification of infected animals was also evaluated.

**Methods:**

Twenty-eight piglets were used. C-reactive protein (CRP), haptoglobin (Hp), serum amyloid A (SAA) and pig major acute-phase protein (Pig-MAP) concentrations in serum were measured using commercial ELISAs.

**Results and Conclusions:**

No relevant clinical signs were observed in intranasally infected pigs. In contrast, coughing, nasal discharge, and fever were observed in pigs infected intratracheally. All infected pigs exhibited specific antibodies in the serum at 10 dpi, and viral shedding was confirmed. The concentrations of CRP, Hp and SAA were significantly increased after infection. The level of Pig-MAP remained constant during subclinical and clinical infection. The concentrations of CRP, Hp and SAA were higher in pigs with clinical disease. Although not specific, strategic APP measurements may reveal ongoing clinical and subclinical infection. A close relationship between the magnitude of serum APP response with the severity of disease, providing an objective tool for validation the severity of infection. The maximum concentration of SAA in serum was closely correlated with lung score and makes this APP potential indicator for disease progress or estimation of treatment strategy.

## Introduction

The acute-phase response is an innate, non-specific immune response which occurs after many different stimuli such as infections, tissue damage, neoplastic growth, or immunological disorders.^1^–^4^ The innate immune response induced by viral infection in the upper respiratory tract is characterized by activation of peripheral primary effector cells that function to initiate a local inflammatory response and recruiting activators of the cellular immune response.^5^ Macrophages present in the respiratory tract produce pro-inflammatory cytokines, for example interleukin -1, interferon, tumor necrosis factor – α causing alterations in local vascular walls, and providing recruitment and activating stimuli to antigen-presenting cells and phagocytes.^5^ Locally produced pro-inflammatory cytokines also cause alterations in hepatic metabolism, inducing the production of various acute-phase proteins (APPs) in the circulation.^6^ The acute-phase response also includes neurological, metabolic and endocrine changes giving rise to leukocytosis or fever development.^3^,^7^,^8^

Swine influenza (SI) is a highly contagious, important respiratory disease, and the main causative viruses are H1N1, H3N2, and H1N2.^9^–^12^ Typical SI outbreaks are characterized by a rapid onset of high fever, loss of appetite, labored abdominal breathing, and coughing.^9^,^10^ Diagnosis of SI infection is currently based on the observation of clinical signs, confirmed by the identification of the infectious agent (PCR, isolation). However, the infection is much more frequent than the disease. Infection with swine influenza virus (SIV) is frequently subclinical, and typical signs are often demonstrated in only 25–30% of a herd.^13^,^14^ Whereas the APP response has been documented for a range of clinical infections, the potential uses of APP for recognition of subclinical infection still remain to be established. It is known that subclinically infected pigs often play an important role in pig-to-pig disease transmission.^8^ Additionally, these animals represent a hazard not only for uninfected animals but also to farmers or abattoir workers.^15^–^17^ Additionally, subclinical infections may reduce growth of pigs and predispose to infection with secondary pathogens.

Only a few studies have investigated the kinetics of APP response in pigs during SIV infection (mostly clinical) caused by H1N1 and H1N2 subtypes.^18^–^21^ Therefore, the objective of this study was to investigate and compare the kinetics of acute-phase protein response during subclinical and clinical influenza in pigs caused by H3N2 SIV. Moreover, the correlation between concentrations of investigated APPs in serum and disease severity was analyzed. Additionally, the usefulness of C-reactive protein (CRP), haptoglobin (Hp), serum amyloid A (SAA) and/or Pig major acute protein (Pig-MAP) measurements in identification of actually infected animals was evaluated.

## Materials and methods

### Animals

Twenty-eight piglets of similar genetics (PIC), approximately 6 weeks old, both sexes, were sourced from high health status herd and prior to the start of the study were shown to be both influenza A virus and antibody (subtypes H1N1, H1N2, H3N2) negative by matrix (M) gene real-time RT-PCR and hemagglutination inhibition assay (HI), respectively. The herd was seronegative to porcine reproductive and respiratory syndrome virus and pseudorabies virus. No evidence of pleuropneumonia, streptococcosis and atrophic rhinitis was recorded at any age group of pigs, based on clinical, serological and pathological examinations.

Animals were divided randomly into four groups: two control groups mock-infected intranasally (IN) or intratracheally (IT) (IN-control and IT-control; *n* = 4 each) and two experimental groups: IN–-infected and IT-infected (*n* = 10 each).

During the experiment, piglets were housed at the BSL3 animal facility of the National Veterinary Research Institute (Puławy, Poland) in independent, isolated units, two for the control pigs and two for the infected pigs. Feed and water were offered *ad libitum*.

Animal use and handling protocols were approved by local ethical commission (University of Life Sciences in Lublin, Poland).

### Preparation of virus inoculum

Swine influenza virus Sw/Ghent/172/2008, subtype H3N2 (hereafter referred to as SwH3N2) kindly provided by Laboratory of Virology, Faculty of Veterinary Medicine, Ghent University, was used for the experimental infection. The stock used for inoculation represented the third passage in eggs. The virus concentration was evaluated in Madin–Darby canine kidney (MDCK) cells and stored at −80°C until used.

### Experimental design

On day 0, piglets from experimental groups (IN-infected and IT-infected) were inoculated with SwH3N2. Inoculations of 1·5x10^7·5^TCID_50_ of virus in 3 ml of phosphate-buffered saline (PBS) were given IN or IT. Eight mock-inoculated pigs (with PBS) served as control pigs.

To examine the events taking place at the early stages of infection with SwH3N2, two infected and one control piglets from respective groups were euthanized on 2 and 4 days post-inoculation (dpi). All remaining pigs in each group (6 infected and 2 controls) were euthanized on 10 dpi. Necropsy was performed immediately after the animals were euthanized.

### Clinical and pathological examination

Rectal temperatures were assessed daily, and clinical signs of disease were recorded. Fever was defined as a rise in body temperature to 40°C or above. The pigs were observed clinically several times a day from the day of infection and throughout the experiment.

Blood samples were collected on days −7, 0 (challenge), 1, 2, 3, 5, 7, and 10 dpi. Serum was harvested after centrifugation and stored at −80°C for further analyses. Nasal swabs were taken at 2, 3, 4, 5, 7, and 10 dpi. Complete necropsy was done on each animal, with special emphasis on the respiratory tract. Gross lung lesions were assessed for the presence or absence of pulmonary cranioventral multifocal consolidation, and when present, extension was recorded. Samples from lung (all lobes separately) and tracheas were collected for viral RNA extraction.

#### Lung Score

Lung lesions were scored using the method developed by Madec and Kobisch^22^ according to the following scheme: point 0, no lesion; point 1, lesions affecting <25% of the lobe surface; points 2, lesions affecting 25–49% of the lobe surface; points 3, lesions affecting 50–74% of the lobe surface and points 4, lesions affecting >75% of the lobe surface. All recorded scores were then added together, to determine final visual lung score for each pig, ranging from 0 to 28.

### Laboratory examination

#### Swabs and tissue samples

The general swine influenza A real-time RT-PCR method was used for detection of SIV in swabs and tissues, as described previously.^23^ Samples with Ct value less than 30 were considered to be M gene positive; samples having Ct value 30–35 with sigmoidal/logarithmic appearance were considered to be weak positive; samples with Ct value more than 35 were considered to be negative.

#### Serum analyses

##### Hemagglutinin inhibition assay (HI)

Antibodies against SIVs were measured using a HI assay. The HI assay was performed according to the standard procedure, using 0·5% chicken erythrocytes and 4HA units of strains H3N2 used for inoculation (SwH3N2) and additionally to check the immune status of the pigs before inoculation with H1N1 (A/sw/Poland/KPR9/2004) and H1N2 (A/Sw/Granstedt/2004). All sera were tested in serial twofold dilutions, starting at 1:20. For estimates of the antibodies prevalence, titers equal or higher than 20 were considered positive. For statistical analyses, titers lower than 20 were set to 1.

##### APP determination in serum samples

For determination of APP, commercial ELISAs were used according to the manufacturer's recommendation (Pig C-reactive protein ELISA and Pig haptoglobin ELISA from Life Diagnostics, Inc., West Chester, PA, USA; PigMAP KIT ELISA from PigCHAMP Pro Europa S.A, Segovia, Spain; Phase Serum Amyloid A Assay and Haptoglobin Kit from Tridelta Development Ltd, County Kildare, Ireland). For all analyses, serum samples were tested in duplicate. Prior to analyses, serum samples were diluted as follows: 1:1000 for CRP, 1:35 000 for Hp, 1:500 for SAA and 1:1000 for Pig-MAP. The APP results are reported on a concentration per ml basis.

### Statistical analysis

The obtained data were subjected to the W. Shapiro–Wilk's test of normality and the Levene's test of equal variances. Comparisons between infected and control groups at each time point were assessed using the Mann–Whitney U-test. For analysis of correlation between measured parameters, the Spearman's rank correlation (nonparametric) was used. Differences with α <0·05 were considered as significant. All calculations were performed with the statistica 8.0 (Statsoft, Cracow, Poland) computer program.

## Results

### Clinical signs

**Figure 1:**
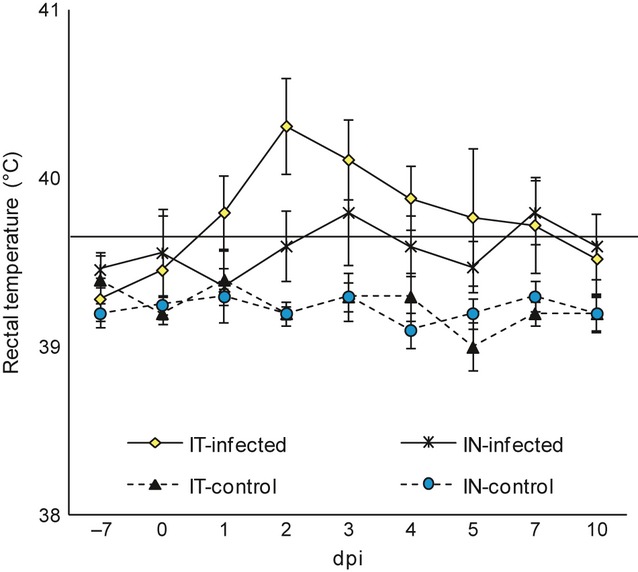
The mean (±SD) rectal temperature of pigs infected with swine influenza type A subtype H3N2 virus and controls, IT,intratracheally, IN,intranasally.

No relevant respiratory or systemic clinical signs were observed in pigs from IN-infected group. However, two IN-infected pigs showed transient fever (40·0–40·3°C) between day 2 and 4 post-infection (dpi). In contrast, coughing, sneezing, nasal discharge, and fever were observed in all IT-infected piglets (typical clinical course of swine influenza). In the control pigs, no clinical signs of any disease were seen and rectal temperatures were below 40°C. The mean rectal temperature of pigs from all groups is shown in Figure 1.

### Antibody response against SwH3N2

All infected pigs exhibited antibodies against hemagglutinin (anti-HA) in the serum at 10 dpi. Piglets infected IT had higher HI titer, and antibodies were detected earlier than in pigs infected IN (from 5 dpi in 1/10 and from 7 dpi in 10/10 of IT-infected pigs). Sera from pigs in the control group had no antibody titers (<20 HI titer).

### Presence of SIV in swabs and tissues

Real-time RT-PCR assay, used to confirm the presence of SIV in the nasal swabs, revealed positive results from all infected pigs between 2 and 5 dpi (in both infected groups) (Table 1). Some infected pigs shed virus also at 7 dpi. In the nasal swabs taken before inoculation, and in the swabs taken from the control pigs, no SIV genetic material was found. In all infected pigs, euthanized at 2 and 4 dpi, the presence of SIV was confirmed in the respiratory tract (mostly in the tracheas and right medium lobes). No viral RNA was detected in respiratory tract on day 10 post-inoculation.

**Table 1:** Real-time RT-PCR results for clinical samples (nasal swabs) of pigs IN- and IT-infected with H3N2 swine influenza virus and non-infected pigs

Animals	0	1	2	3	5	7	10
Times post-IN inoculation (days)							
1	−	++	++	++	+	+	−
2	−	+	+	++	++	−	−
3	−	+	++	++	+	+	−
4	−	+	++	++	+	+	−
5	−	−	+	++	+	−	−
6	−	+	++	++	++	−	−
7	−	−	+	++	n/a	n/a	n/a
8	−	+	+	++	n/a	n/a	n/a
9	−	+	++	n/a	n/a	n/a	n/a
10	−	++	++	n/a	n/a	n/a	n/a
Times post-IT inoculation (days)							
1	−	++	++	++	++	++	−
2	−	+	++	++	++	+	−
3	−	++	++	++	++	+	−
4	−	++	++	++	++	+	−
5	−	++	++	++	+	−	−
6	−	++	++	++	++	−	−
7	−	++	++	++	n/a	n/a	n/a
8	−	+	++	++	n/a	n/a	n/a
9	−	++	++	n/a	n/a	n/a	n/a
10	−	++	++	n/a	n/a	n/a	n/a
Non-infected pigs							
All piglets	−	−	−	−	−	−	−

Real-time RT-PCR results are given as ++ (Ct value <30; positive), + (Ct value 30–35; weak positive), – (Ct value >35, negative), n/a, not applicable.

### Pathological examination

**Figure 2:**
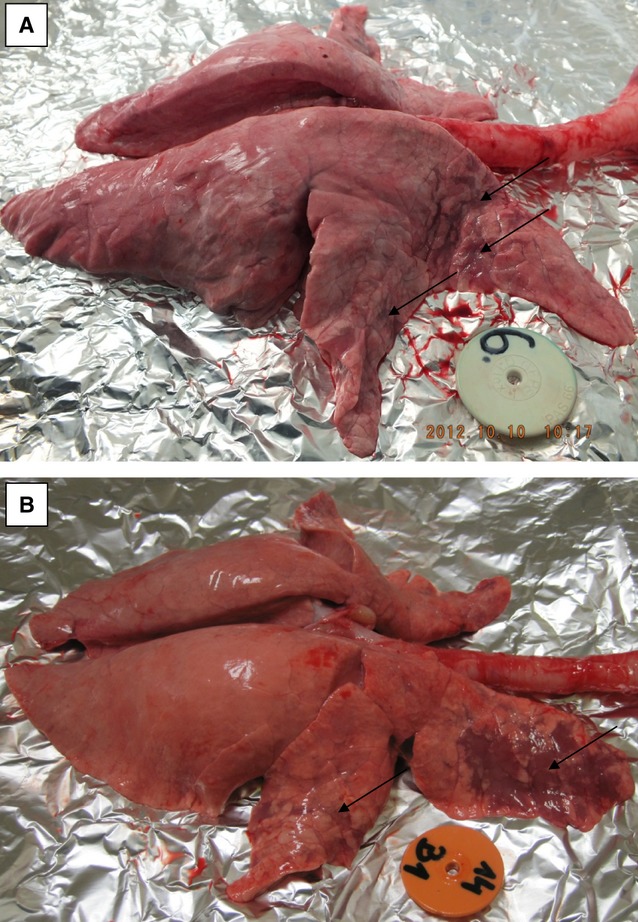
Typical pathological changes observed in the lungs of pigs infected with swine influenza virus (H3N2) intranasally (A) or intratracheally (B).

Post-mortem examination revealed typical lesions deriving from SIV infection in the lungs of 8/10 infected pigs from IN-infected group and in 10/10 from IT-infected group. One IN-infected pig euthanized at 2 dpi and one euthanized at 10 dpi had no macroscopic changes in lungs. The typical lesions observed in the lungs of infected pigs are shown in Figure 2. The mean lung score was 2·80 ± 1·81 (range 0–5) for IN-infected group and 4·10 ± 2·39 (range 1–7) for IT-infected pigs.

### Acute-phase proteins

**Figure 3:**
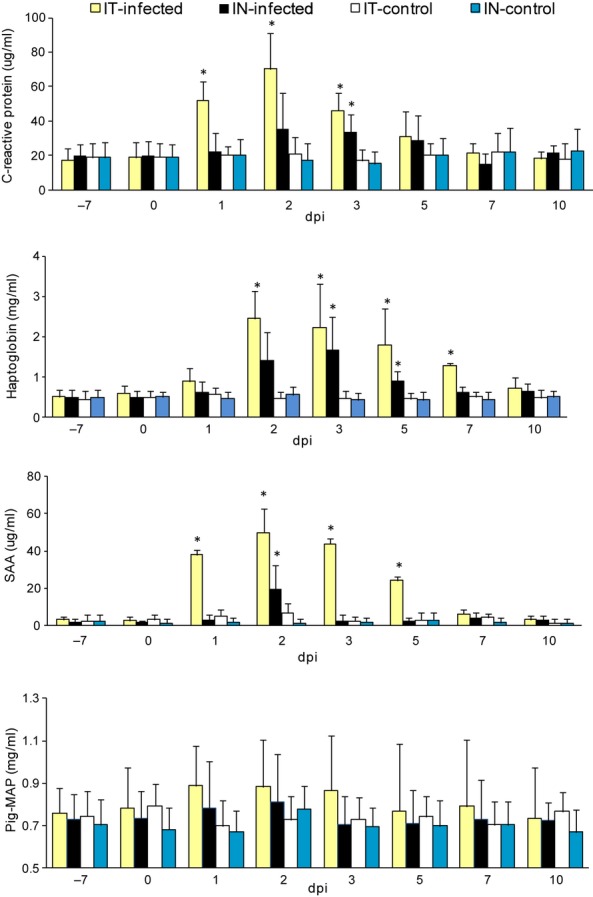
The mean (±SD) concentrations of CRP, Hp, SAA and Pig-MAP in serum of pigs before and after IN and IT inoculation with H3N2 swine influenza virus, and in the control groups. *Differences significant as compared to respective control group (*P* < 0·05).

The time course of mean CRP, Hp, SAA and Pig-MAP concentration (±SD) in infected and control pigs during the study period is shown in Figure 3. In pigs infected IT, the mean concentration of CRP was significantly higher from 1 to 3 dpi, as compared to the control (*P* < 0·05). The mean maximum concentration reached 70·33 ± 22·57 μg/ml and was almost fourfold higher than before inoculation. Significant rise in CRP concentration was also observed in pigs with subclinical course of SI (IN-infected) but only at 3 dpi (*P* < 0·05). The mean maximum concentration was approximately twofold higher as compared to day 0.

Haptoglobin shows pre-infection levels of 0·65 mg/ml or below in all animals. In IT-infected piglets the mean concentration significantly increased from 2 dpi and remained elevated to 7 dpi (*P* < 0·05). The mean peak level of over 4 times the day 0 mean level was seen at 2 dpi. Regarding Hp response during subclinical influenza, the response was weaker (about threefold increase), shorter and was observed later (from 3 to 5 dpi) after infection as compared to clinical influenza.

Serum amyloid A showed pre-inoculation levels of 7 μg/ml or below in all animals. After IT infection SAA level rose sharply in all pigs on day 1 pi and peaked day 2, reaching mean peak level of around 20 times the mean level of before inoculation. The significantly higher concentration of SAA was observed to 5 dpi (*P* < 0·05). In contrast, in piglets with subclinical course of the disease, the significantly higher concentration was observed only at day 2 pi, reaching mean peak level of about 12 times the day 0 level. In the control pigs, levels of all investigated APP remained relatively constant.

#### Acute-phase protein responses – correlation with clinical findings

The concentrations of CRP, Hp, and SAA were generally higher in pigs with clinical course of influenza. Moreover, the significantly higher levels of these APPs were observed for longer period of time, as compared to subclinically infected pigs. The rise in rectal temperature coincided with the changes in CRP, Hp, and SAA concentrations. Significant correlation was found between maximum concentration of SAA in serum and changes in the lungs with regard to both clinical and subclinical course of the influenza (R-Spearman = 0·77 and 0·97, *P* < 0·05, respectively).

## Discussion

In present study, early response of CRP, Hp, SAA, and Pig-MAP induced by IN and IT infection with SwH3N2 virus was characterized and compared, during the first 10 dpi. In addition, the relationship between APPs concentrations and pathological changes in the lungs were analyzed. Measurement of serum concentrations of CRP, Hp, and SAA indicated a rapid acute-phase response in IT-infected pigs, which coincided with the appearance of clinical signs of disease. The finding that the rise in rectal temperature coincided with changes in CRP, Hp, and SAA in serum is to be expected, as all these parameters reflect the onset of the systemic acute-phase response to infection.^5^ After IN infection, no relevant clinical signs were observed, and APP response was weaker than during clinical influenza. In accordance with our previous reports, the concentration of Pig-MAP remained unchanged in both infected groups.^20^,^21^ No response of Pig-MAP was observed also in other viral disease in pigs (porcine reproductive and respiratory syndrome, pseudorabies).^24^

The APP responses to experimental infections with other subtypes of SIV have previously been studied by Barbé *et al*.^19^, Brookes *et al*.^18^ and Pomorska-Mól *et al*.^20^,^21^. An increase in CRP and Hp concentrations was reported by Brookes *et al*.^18^ during acute infection caused by pandemic H1N1 2009 virus. The CRP levels peaked at 4 dpi, while Hp responses were more protracted, peaking at about 10 dpi. Different kinetics of the APP response observed by Brookes *et al*.^18^ could be the results of different virulence of virus used for experimental infection. In our study, the concentration of CRP and Hp increased also during subclinical infection, but the significant increase was observed earlier and for shorter period of time.

The acute-phase proteins associated with acute SI in pigs were also investigated by Barbé *et al*.^19^ during first 5 dpi after inoculation of pigs with SIV H1N1 strain. In general, the serum concentrations of CRP and Hp peaked at 2 dpi, but no significant differences were found between infected and control pigs. However, it could be a result of small number of pigs under study of Barbé *et al*.^19^.

The knowledge of SAA response in viral diseases, including SI, is limited. Increase in serum SAA has been reported in human and horses infected with influenza virus.^24^,^25^ Whicher *et al*.^24^ found that in human after experimental influenza A virus infection, SAA and CRP concentrations increased and reached their maximum concentrations on day 3 after viral challenge. Moreover, in agreement with our findings, the significant increase in SAA was observed also in subjects that did not develop clinical signs but did excrete virus. Hulten *et al*.^25^ found that equine SAA responds to equine influenza infection and that a positive correlation was found between concentration of SAA in horses and clinical signs during disease. Our results are in agreement with these findings.

Similar findings, with regard to the response of all investigated APP in IT-infected pigs, were previously observed by us after experimental infection with H1N2 SIV. In infected pigs, concentration of CRP, Hp, and SAA increased significantly at the time when the most amount virus was shed (from 1 to 3 dpi) and the level of Pig-MAP remained unchanged to the end of study.^21^

Until now, only one report has been published on the development over time of acute-phase response during subclinical infection with SIVs.^20^ In the present study, no significant clinical signs were observed in any of the IN-infected pigs; however, all IN-infected animals developed specific anti-HA antibodies against SwH3N2, and viral shedding was observed from 2 to 7 dpi. Most investigated APPs were significantly elevated, with exception of Pig-MAP. However, the APP response was weaker and less protracted, as compared to response observed in pigs with clinical influenza. Only concentration of Hp was elevated significantly for more than 1 day (from 3 to 5 dpi). Similar findings were observed previously during subclinical infection with H1N1 influenza virus in pigs.^20^ In H1N1 model, only Hp and SAA were significantly induced while concentration of CRP and Pig-MAP remained generally unchanged.^20^ Although in half of the infected pigs the concentration of CRP tended to increase at 1 dpi, it was not of statistical significance.

To our knowledge, this is the first time that APPs have been evaluated and compared in experimental clinical and subclinical infection with SwH3N2. Observations from our experiments indicate that combined measurement of two or three APPs with different response characteristic; for example, Hp, SAA (increased concentration), and Pig-MAP (unchanged concentration) would give a higher sensitivity of detection throughout the course of ongoing clinical, but also subclinical, SIV infection. Thus, measurement of these APPs in serum can be used as markers of acute systemic response to H3N2 infection in pigs. This could, for example, be valuable during vaccine studies or transmission experiments with SIV isolates of low virulence, where it would be of interest to detect any systemic immune response in animals exposed to the virus. Although not specific, strategic APP measurements in the pig herds may help to reveal ongoing infection, also subclinical, enable pinpointing of infected animals.

Moreover, the results of present study indicate a close relationship between the magnitude of serum APP response with the severity of disease, providing an objective tool for validating the severity of infection. The maximum concentration of SAA in serum was closely correlated with pathological lesions typical for SI and makes this APP potentially important indicator for disease evolution or estimation of treatment strategy. Though, to confirm this hypothesis, more studies are needed, especially under field conditions.
